# Design principles of a computer controlled multiplexed absorptiometer for reaction rate analysis

**DOI:** 10.1155/S1463924679000249

**Published:** 1979

**Authors:** M. Snook, A. Renshaw, J. M. Ridcout, D. J. Wright, J. Baker, J. Dickins

**Affiliations:** 1 Division of Clinical Chemistry Clinical Research Centre Harrow Middlesex HA1 3UJ UK; 2 Department of Clinical Chemistry Northwick Park Hospital Harrow Middlesex HA1 3UJ UK; 3 Division of Bioengineering Clinical Research Centre Harrow Middlesex HA1 3UJ UK


### Design principles of a computer controlled multiplexed absorptiometer for reaction rate analysis

**M. Snook, A. Renshaw, J. M. Ridcout**
*Division of Clinical Chemistry, Clinical Research Centre, Harrow, Middlesex HA1 3UJ.*

**D. J. Wright**
*Department of Clinical Chemistry, Northwick Park Hospital, Harrow, Middlesex HA1 3UJ.*

**J. Baker, J. Dickins**
*Division of Bioengineering, Clinical Research Centre, Harrow, Middlesex HA1 3UJ.*

IN clinical biochemistry laboratories, the importance of quantitative enzyme assays and enzyme-linked analyses steadily increases, and the merits of kinetic methods for monitoring these reactions are well recognised [1]. Reviews of the kinetic aspects of analytical chemistry [2] and of the applications of enzymes in analytical chemistry [3] indicate the need for absorptiometers which are able to monitor reactions continually over increasingly long periods of time. The performance of current instrumentation, however, is limited [4]; either the time during which reactions are followed must be limited in order to maintain an acceptable sample throughput, or the sample throughput restricted in order to obtain suitable reaction times.

This paper describes the design of an analytical system which overcomes these two limitations. The system offers fast sample throughput, together with long observation times and good data resolution. The basic design principle is the sequential monitoring of a large number of cuvettes in a circular array, by means of a single multiplexed absorptiometer. The yield of data is determined by the time taken for the monitoring system to return to an individual cuvette, i.e. the scan rate and the total residence time of a sample in the system. The residence time of a sample is in turn determined by the total number of cuvettes and the specimen throughput, corresponding to the frequency with which a new sample is introduced. The actual values of these parameters can be determined when a particular instrument system is configured. Thus, for a practicable design of instrument, if there were an array of 100 cuvettes, and a new sample were to be introduced every 24 seconds, then the maximum possible observation time could be 100 x 24 seconds = 40 minutes, with a specimen throughput of (60 x 60)/24 = 150 per hour. The scan rate would be totally independent of the two parameters, so that if, for example, an individual cuvette were monitored at 2 second intervals, then in a period of 40 minutes, the sample would yield (40 x 60)/2 = 1200 data readings, and the whole system, with 150 samples, would generate 150 x 1200 = 180,000 readings per hour.

Because of the large yield of analytical data, it is necessary to integrate a computer into the overall design, chiefly for data

*Figure 1 General arrangement of scanning absorptiometer. Samples are diluted during transfer from sample tray T, by transfer arm A in the reaction/optical cuvettes on the analytical rotor, AR. The scanning beam B, is shown just entering the reading sector. The analytical rotor indexes forward 3.6° every 24 seconds, carrying within it the continuously scanning optical beam. Cuvette cleaning, sample transfer, and reagent addition occur within the non-reading sector.*

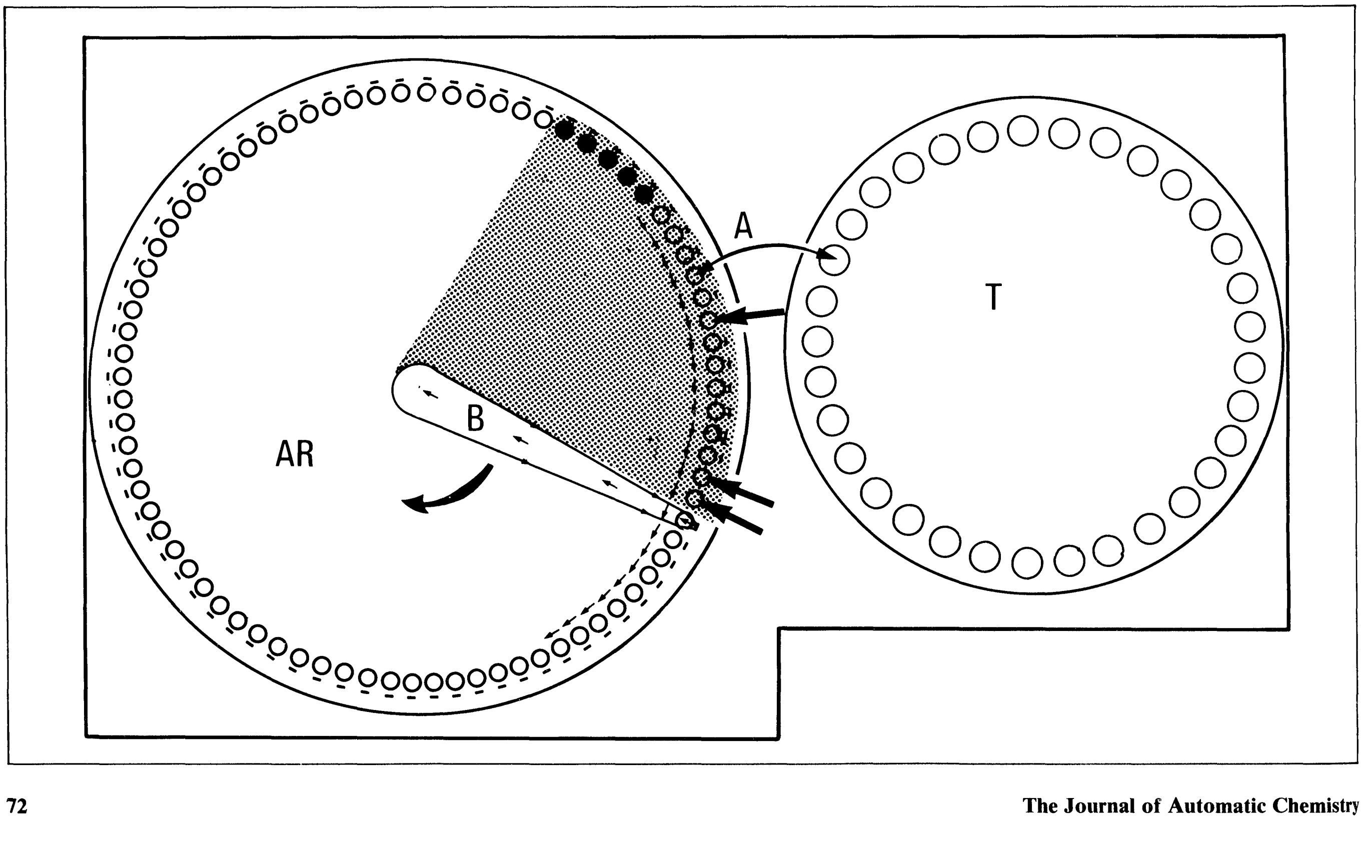

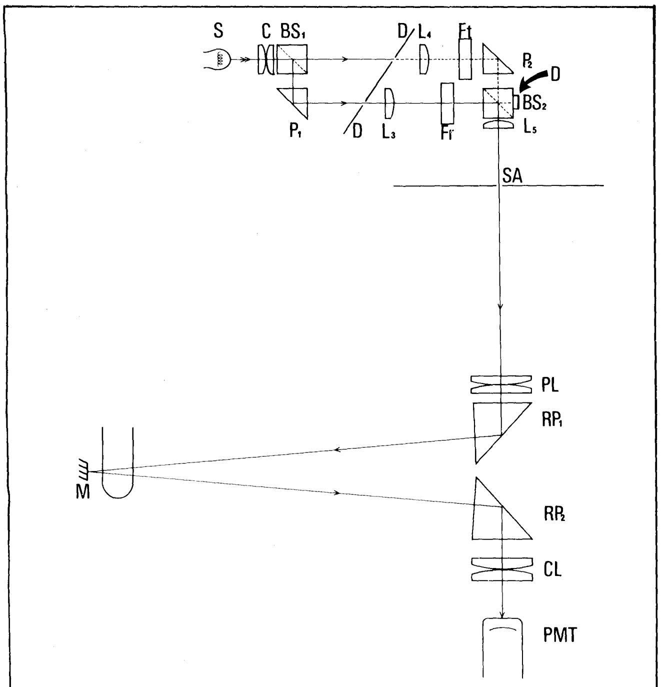

*Figure 2 Optical System. Refer to text for legend.*

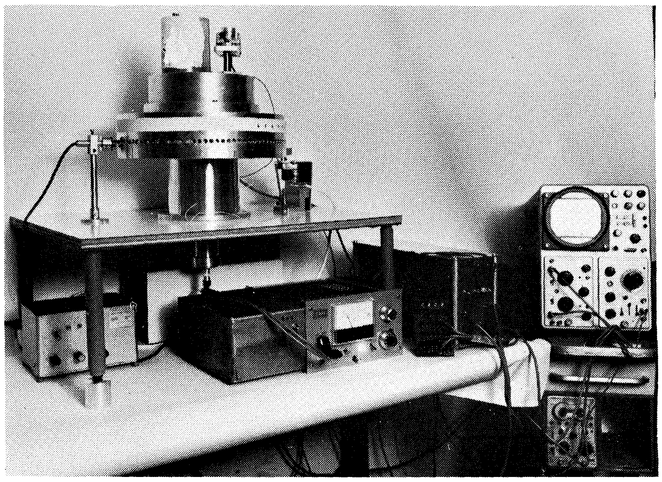

*Figure 3 Photograph of prototype instrument showing the relative positions of scanning optics and cuvette turntable.*

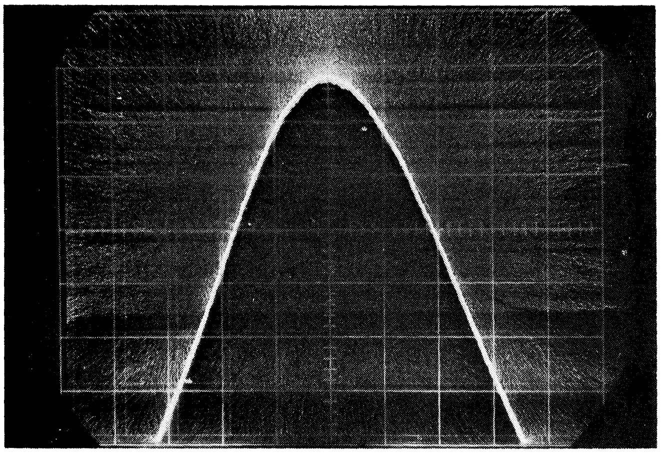

*Figure 4 PMT signal from single cuvette at single wavelength during 20 m sec scan.*

handling, but also, in part, to function as a process controller. A Texas 960A computer was used in this work.

The design of the multiplexed absorptiometer, the configuration of the associated electronics and the general software philosophy are now described in some detail since it is these features, rather than the sample processing and reagent addition mechanisms which are novel.

### Measurement principles

In an ideal absorptiometer, the output signal would be a function of the ratio of the radiant power transmitted by a cuvette filled with absorbing solution, to the radiant power transmitted by an identical cuvette filled with solvent or blank solution, as measured simultaneously on two photometers having identical characteristics. However, this is not practicable in design terms, so that in conventional absorptiometers, this ideal is approximated by using two precision cuvettes in the test and reference optical paths respectively, both being part of a common photometric system. Readings may be taken alternately in rapid succession, for example, with choppers run at 400 Hz, and the ratio between the two channels thus obtained. This 'reference cuvette' approach [5] is also acceptable in multi-cuvette instruments (such as GeMSAEC), originally described by Anderson [6], providing that all of the cuvettes are matched to reasonably close tolerances, that calibrating runs are made to establish correction factors to counteract any cuvette-to-cuvette differences, and that measurements are normalised to a reagent blank reading that is updated at a rate of 40 Hz or more.

However, in the present scanning beam design, where 100 cuvettes are to be scanned every 2 seconds, normalization at this relatively slow rate of scanning would not be acceptable if the rates of change of absorbance value is to be measured accurately. A practical alternative is to measure all absorbance values at two wavelengths. With proper selection of the secondary (reference) wavelength, the reference reading can give a measure of the radiant power transmitted by the solvent or blank solution, with which the radiant power transmitted by the test solution can be compared.

Ten test and ten reference measurements are taken alternately on every cuvette during each pass of the scanning beam to reduce mechanically induced noise on the photometric signal, and minimise any bias in the signal acquisition at the chosen scanning rate of 0.5 Hz. The ten readings at each wavelength are integrated for each cuvette. This approach approximates the ideal design, in that the same photometer and cuvette are used for test and reference readings. The only departure from the ideal is that measurements are made alternately, rather than simultaneously, but for the relatively slow reactions which this system is particularly designed to monitor, the changes of absorbance during the 10 m sec when the signal is acquired from individual cuvettes are negligibly small. The optical, mechanical, and signal acquisition systems discussed in the following sections illustrate how the measurement principles have been implemented, and describe how the software optimizes the overall performance of the system.

### Optical design

The general arrangement of the scanning absorptiometer is shown in Figure 1. Specimens are loaded on to a sample tray, and samples are aspirated and diluted into cuvettes on the rotor by the sample transfer arm. At the time the sample is diluted, reagents are added through appropriately positioned reagent dispensers to previously diluted sample. The sample tray and analytical rotor then both index forward one position, ready to repeat the cycle of sample dilution and reagent addition. The optical layout of the instrument shown in Figure 2 comprises two sections — a dual channel monochromator, and the scanning optics. The function of the monochromator is to generate two wavelengths of exciting radiation with similar geometric characteristics, and the scanning optics are designed to direct the output of the monochromator sequentially to all the cuvettes and to recover the reflected signal.

The inter-relationship between the optics and the mechanical components is illustrated by Figure 3 which is a photograph of the prototype instrument built in these laboratories.

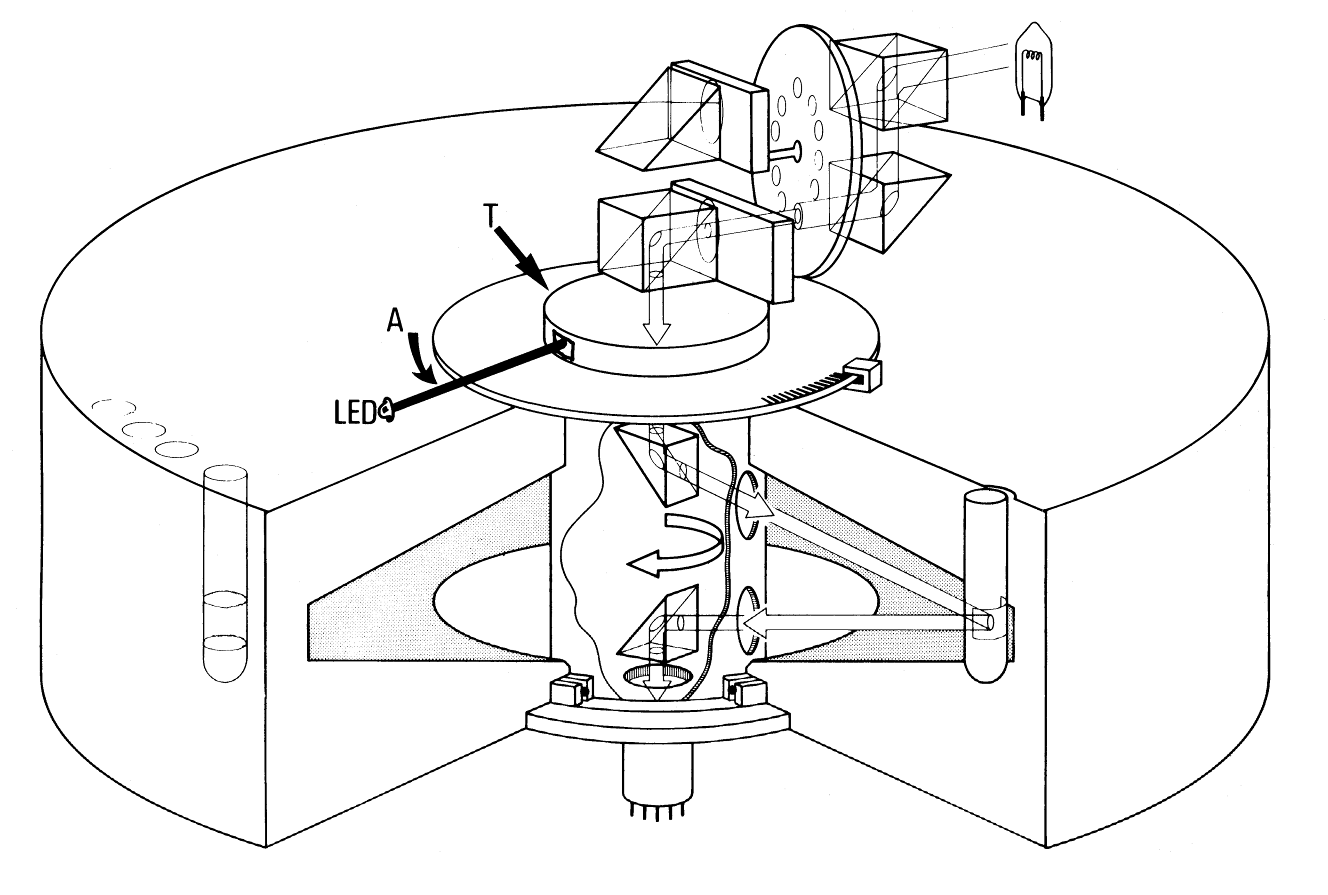

*Figure 5 General optical and mechanical layout. Refer to text for legend.*

In the monochromator, radiation from the tungsten halogen lamp S is collected by a pair of condenser lenses C onto the beam splitting cube $BS_1$. Light reflected in the cube is further reflected by the prism $P_1$ to form the reference beam parallel to the transmitted beam in the test channel. The chopper disc D is so designed and positioned that the test and reference beams are interrupted alternately. The appropriate interference filters $F_t$ and $F_r$, are placed between collimating lenses $L_3$ and $L_4$ and the beam combining cube $BS_2$. After monochromation, the beam in the test wavelength channel is reflected downwards by prism $P_2$ and combined with the reference channel in the cube $BS_2$. The chopped beam is finally focused on the source aperature SA by the lens $L_5$. As beam splitters are only 50% efficient in the recombining mode, a photo-detector D is cemented to the unused face of $BS_2$, and in conjunction with appropriate comparator circuitry is used to define the wavelength output of the dual channel monochromator. The two reflective prisms $RP_1$ and $RP_2$ provide the scanning motion, being mounted within the tube of a precision optical shaft encoder, which in addition to providing the positional information required for data acquisition, allows the precision bearings of the encoder to be used to carry the scanning beam.

In conjunction with prism $RP_1$, the projection lenses (PL) project an image of the source aperture (SA) on to a concave mirror (M) located behind each cylindrical cuvette. The resulting image reflected by M is focused by the collecting lenses (CL) on to the photomultiplier tube cathode (PMT).

The cuvette and its contents are a functional part of the system, in that together they act as a cylindrical lens of short focal length. The gathering power of this lens and of the reflecting concave mirror is such that, during scanning, the image focused onto the PMT cathode by the collector lenses CL is 'stretched', i.e. the scanning beam can swing through an arc of almost 1°, and still maintain a useable image of the illuminated cuvette on the PMT (Figure 4). This advantage is not realised if a scanning beam of parallel light illuminates a square section cuvette, and is reflected by a plane mirror. Such an arrangement results in a signal of only a few micro seconds duration at the time that the beam is normal to the reflecting mirror. Also in this situation the optical alignment is critical and it is very difficult to achieve the necessary response time from the process control minicomputer used.

### Mechanical Design

The general arrangement of the absorptiometer with 100 optical cuvettes radially dispersed about the vertical axis of the reaction rotor is shown in Figure 5. The optical components of the dual channel monochromator are rigidly mounted within a machined aluminium housing (which is not shown in the diagram), and the housing itself is solidly fixed to the top of the rotor.

The precision optical shaft encoder accurately relates (to within 0.04°) the signals from the photomultiplier to the appropriate cuvettes. The shaft encoder tube, which contains the reflecting prism (Figure 2) is driven by a small synchronous motor and associated gear train; this provides the rotation of the scanning beam.

The photomultiplier and the collecting lenses of the detector optics are also mounted rigidly to the rotor, so that the relative positions of all the optical components, except for the rotating reflecting prisms, are rigidly fixed with respect to each other. In this prototype apparatus, the electrical connections to the incoming power supplies, and the outgoing analogue and logic signals, are made using flexible leads which are hard-wired. For routine investigations slip rings are used to achieve the longer periods of operation necessary.

It is part of the concept of this instrument that the cuvette cleaning, sample transfer and reagent addition mechanisms remain static relative to the equipment mainframe. It is therefore necessary to impose a stepwise movement on the analytical rotor

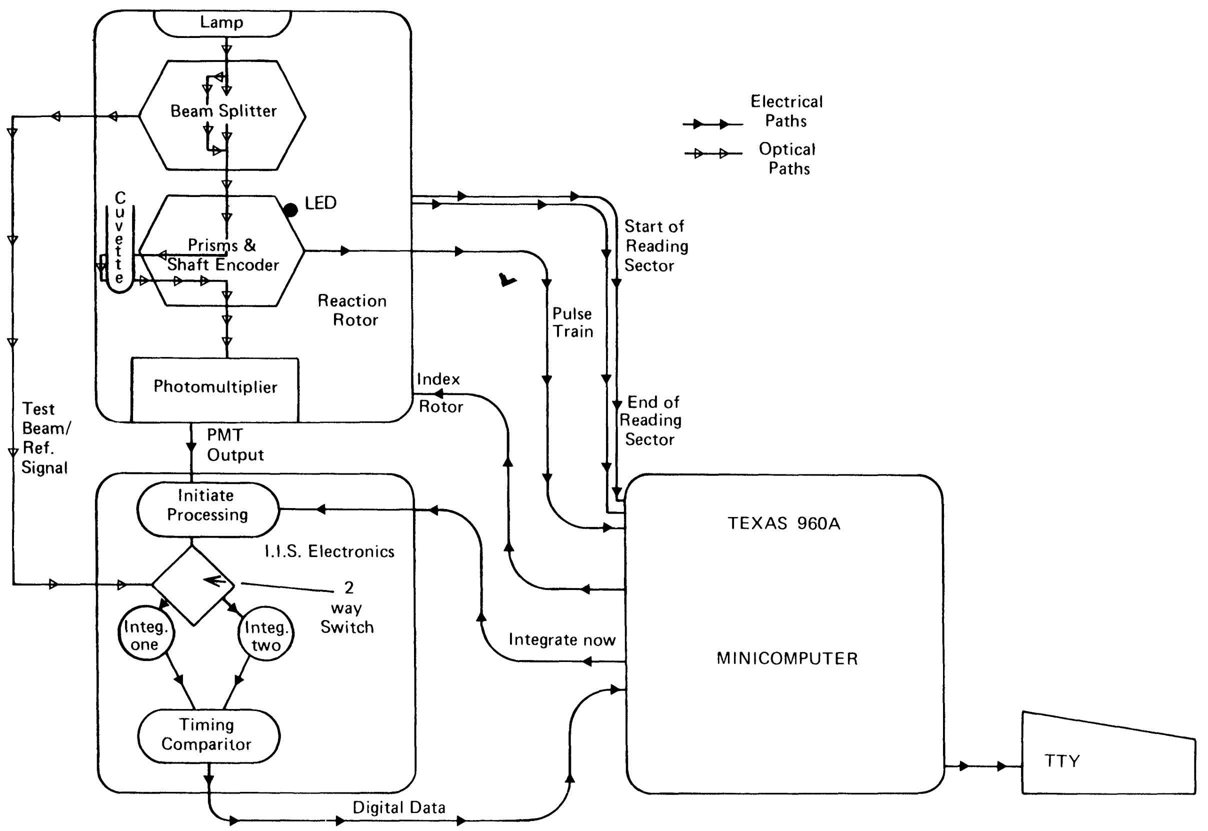

*Figure 6 Signal acquisition and control pathways between the multiplexed absorptiometer and the Texas 960A.*

of 3.6° with respect to the equipment mainframe every 24 secs. Since the rotor carries within itself the continuously scanning beam of the absorptiometer, another monitoring device is required to define the scanning sector, and relate the effect of the double movement of the beam to the mainframe. This is achieved by fitting an LED to the end of the arm A, itself fixed to the rotating encoder tube T; a light shield (not shown), drilled with 100 holes on its circumference, covers this rotating miniature light source, and the passage of the light behind the 100 holes is monitored by two photodetectors fixed to the equipment mainframe. The detectors are not adjustable and always define the maximum reading sector. This low resolution encoder provides the interrupts necessary to define the START and END of the reading sector. The computer software routines recognise these interrupts and marrys the correct data to the cuvettes and avoids any mismatching of the data recorded.

The flexibility required to handle methodolgies where a lag phase must be allowed, or where reagents must be added is incorporated into the software routines. In this manner, any data points which do not meet the specified criteria for the analysis are therefore ignored. Stepping of the reaction rotor to receive a new sample is achieved by a simple rim drive arrangement (not shown) which is activated when the scanning beam leaves the reading sector on the 12th scan. The 3.6° indexing movement is completed in less than 0.5 sec, i.e. before the scanning beam enters the new reading sector to take the 'Time Zero' reading on the cuvette that has just entered, and this ensures that no readings are taken while the rotor is indexing forward.

### Signal Acquisition and Processing

Figure 6 is a schematic illustration of the interrelationship of the functional parts of the analyser, showing the systems controlling signals and the analogue data processing pathways. It is convenient to consider the signal acquisition and processing steps from the cuvette through to the data processing computer.

The gathering power of a filled cuvette and the corresponding concave mirror 'stretch' the signal as the beam scans a cuvette, but accurate initiation of signal acquisition is still necessary. Initiation occurs at a point just before the maximum output of the waveform signal (Figure 4) is reached and is defined by the position of the shaft encoder. The encoder however, does not indicate absolute position, and it is necessary to use the Texas 960A computer to count the encoder pulse train from a fixed reference point. To this end, an accurate zero reference is provided within the encoder itself.

In the prototype instrument described here, the dimensional tolerance between cuvette centres is within $\pm$ 0.05 mm corresponding to $\pm$ 0.04° arc; with this level of precision, the encoder count between adjacent cuvettes is 90 $\pm$ 1, and this simplifies the logic required for signal acquisition. Once a point has been established, as determined automatically by a software routine, that enables signal acquisition on cuvette 1, i.e. the first cuvette immediately following the shaft encoder zero reference, the pulses from the encoder are counted. When a count of 90 is reached, signal acquisition is again enabled and the counter reset to zero. This sequence is repeated until the 100th cuvette has been read, at which time the counter is reset to zero with the encoder zero reference: this precludes the possibility of any cumulative errors due to 'drop outs' or 'noise' in the ongoing count.

The only process control function of the computer with respect to signal acquisition, is the counting task described above. This is achieved via the input/output board, which is normally under the control of a programmable interval timer. In the context of signal acquisition however, it is not timing that is critical, but the rotation of the scanning beam mounted within the encoder. To ensure synchronism in this task, the interval timer was modified and the manufacturer's crystal clock pulse train replaced by the shaft encoder pulse train. In this manner, all of the process control functions are related to the position of the scanning beam and not to time.

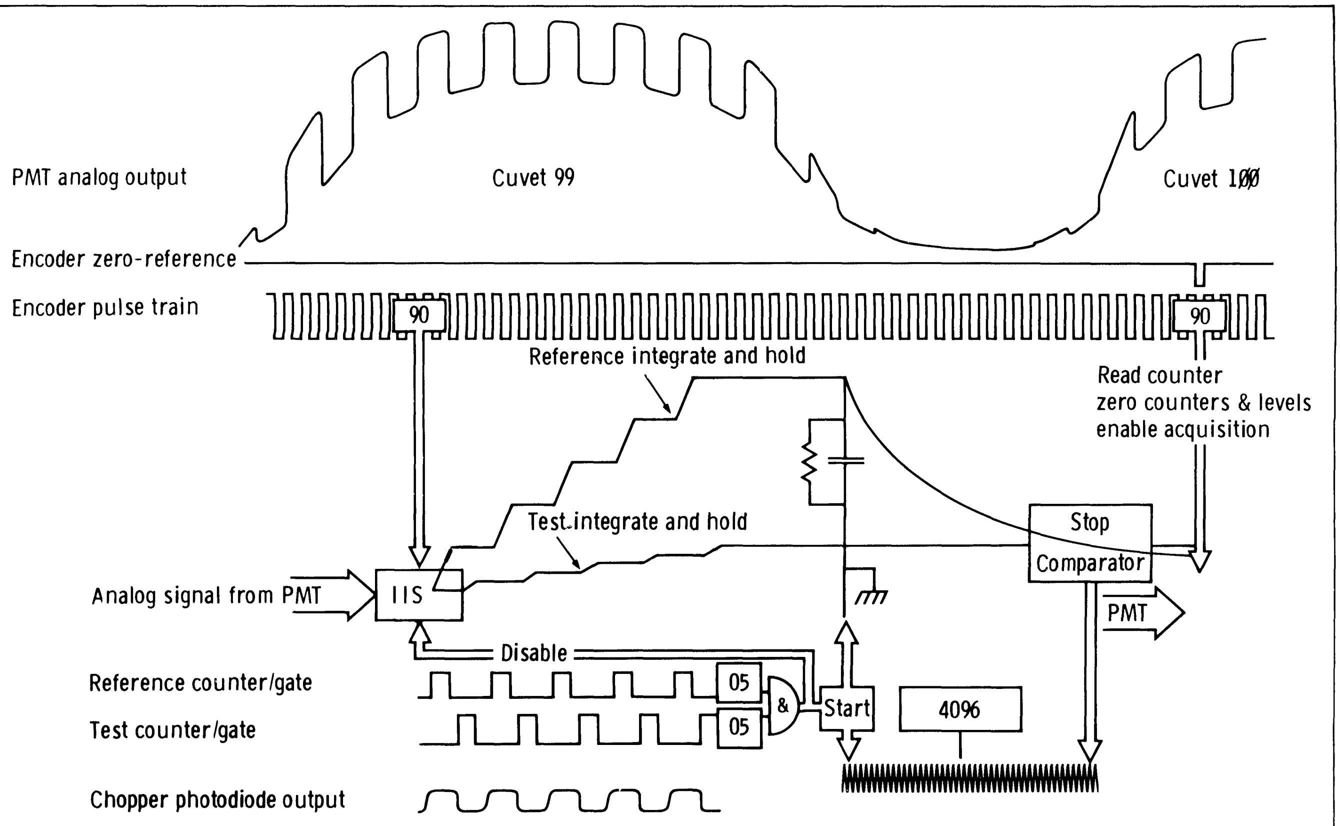

*Figure 7 Inter-relationships of shaft encoder pulse train, PMT output, monochromator chopper photodiode output, signal acquisition integrators, Beer's Law correction, and analogue to digital conversion.*

The scanning beam and the chopper disc are not in synchronism with each other so the chopper photodetector (D, Figure 2) can indicate any one of three states when the signal acquisition is enabled — sample, reference or transition. The relationships between the shaft encoder and pulse train, the shaft encoder zero reference, the chopper photodetector output, and the photomultiplier analogue output, may be explained with reference to Figure 7.

When the beam is in the scanning sector and the encoder pulse train reaches a count of 90, the sample and reference integrator input switch (IIS) is enabled by the counter latching a bistable that is part of the IIS circuit. This counter immediately resets the zero and continues counting. The chopper photodetector switches the photomultiplier signal to the appropriate integrator, and the signal is sampled for a finite period of real time, which is indicated in Figure 7 by the sampling pulse widths being narrower than the chopper photodiode output; this minimises the effects on the integrated signal of any variations in chopper speed or geometry. No signals are sampled during the transition from one wavelength to another.

The number of integrations at both test and reference wavelengths is counted, and when an equal pre-set number is reached, the integrator input switch is disabled to stop further data acquisition. A few micro-seconds later another bistable ('start clock') simultaneously applies an R/C network to the reference integrator output, and starts a high frequency (1 M Hz) crystal clock driving into a counter. The exponential discharge of the reference integrator down to the held sample signal, is monitored by a comparator that resets the bistable 'start clock' when the two signals are the same. The counter now contains a number that is proportional to the absorbance of the sample.

On receipt of the next 'enable acquisition' signal from the shaft encoder counter, the contents of the clock counter which are designated a datum point in the context of software, are read into the computer, the counter reset to zero, and the test and reference integrators momentarily clamped to discharge and residue signal. The entire system is now ready for the data acquisition cycle to be repeated.

### Software Philosophy

The classification of kinetic methods proposed by Pardue [7] is adopted. The defined objective of measurement in this system is to obtain the best regression fit on a minimum of 10 data points, taken over either a fixed time (i.e. the maximum time for slow reactions) or variable time (for reactions complete in less than 34 min which is the maximum practical observation time). In an analytical system generating information at the rate of 50 datum points per second, with reactions being monitored for up to 2040 seconds, effective data reduction is of prime importance. To reduce this large quantity of analytical data to more manageable proportions an algorithm was devised which optimizes the timebase of the measurements on each individual specimen.

When a cuvette first enters the reading sector, the datum from the first pass of the scanning beam is stored in a cyclic buffer as 'Time Zero'. After ten data points have been stored in the cyclic buffer, the change in absorbance ($\Delta A$) between Time Zero and 18 secs, corresponding to the tenth data point, is checked: if $\Delta A$ exceeds a minimum threshold (which is experimentally determined and entered with the software routine), then the regression slope and standard error are calculated, and subsequent readings taken at 2 sec intervals. However, as only 10 datum points are stored for any given reaction, the oldest data point must be deleted before new data are entered. With successive entries of data, both the slope and standard error are recalculated; this process continues until the standard error increases, then the previous slope is taken as having been calculated from the most linear part of the reaction. When the best fit has been determined the cuvette is declared inactive, and no further data collection occurs.

If the minimum threshold is not exceeded after 18 secs, one criteria for 'good data' fails and the program then branches to a different procedure in which alternate data in the cyclic buffer are deleted, the remainder packed down, and new data entered at 4 sec intervals. This process of checking the $\Delta A$ between 'Time Zero' and the tenth data point, and then doubling the time base, can provide reading intervals of 2, 4, 8, 16, 32 and 64 sec on any cuvette. Calculation of the $\Delta A$ value and checking that it exceeds the predetermined minimum occurs when a signal is being acquired on the following cuvette. The advantage of this algorithm is that although the time base for individual reactions has a dynamic range of 64.2 within the overall monitoring period of between 18 sec and 34 min, only 10 data points are stored for any given cuvette.

The calculation routines for regression slope and standard error of reaction for the cuvette that is leaving the reading sector, are time shared with data acquisition prior to that cuvette being cleaned ready to receive a new sample at the sample transfer position. When the scanning beam leaves the reading sector, other computer controlled tasks begin. Printing of results is time shared with the foregoing mathematics package, and with a throughput of a fresh sample every 24 sec, the total time available for calculation and printing is 12 x 0.5 = 6 seconds, corresponding to 12 revolutions of the scanning beam and the time the beam spends in the non reading sector. A further process control task is that of counting the rotations of the scanning beam. This provides the necessary command pulse required every 12th revolution, to index the entire rotor forward on completing one cycle ready to receive the next sample.

### Discussion

The basic principle embodied in this experimental prototype of a scanning multiplexed absorptiometer working within a continuous feed preparative unit, allows for both high sample throughput and a wide range of reaction times. The system as described, with the absorptiometer functioning in a multiplexed scanning mode, can monitor reactions over the range of 18 sec-34 mins, but can be extended — at the expense of sample throughput — to monitor reactions over even longer periods (e.g. 45 min monitoring, at 100 tests per hour). Shorter observation times for fast reactions can be realised by mechanically locking the absorptiometer beam to the cuvette under investigation; this enables a reaction to be monitored from 'Time Zero' to 23 sec. Using the analogue processing techniques described, the data rate is limited to approximately 100 datum points per sec but the algorithm and mathematics package function in the same way as for slower reactions.

The principles of the system will be incorporated in a new instrument under development by Coulter Scientific Inc., Hialeah, Florida, USA. Preliminary results [8] obtained on the prototype were presented at the Tenth International Congress of Clinical Chemistry, Mexico City 1978. It is hoped that a system similiar to that described will become commercially available in the near future, providing the analyst with a high capacity analyser able to monitor reactions over periods longer than those systems currently in use.

ACKNOWLEDGEMENTS

The authors wish to thank Dr. S. S. Brown, Dr. F. L. Mitchell and Mr. S. N. Pocock for their encouragement and advice and would also like to acknowledge the significant programming contribution of Mr. G. Hardcastle.

REFERENCES

[1] Instrumentation Guidelines Study Group. American Association of Clinical Chemists 1977, 23, 2160.
[2] Greinke, R. A., Mark Jr., H. B., *Analytical Chemistry* 1978, 50, 70R Fundamental Reviews.
[3] Fishmann, M. M., *Analytical Chemistry* 1978, 50, 261R Fundamental Reviews.
[4] Tarbit, I., *Laboratory Equipment Digest* 1978, May, p.75.
[5] Avery, J., Gregory IV, R. P., Renow, B. W., Woodruff, T., Malmstadt, H. V., *Clinical Chemistry* 1974, 20, 942.
[6] Anderson, N. G., *American Journal of Clinical Pathology* 1970, 53, 778.
[7] Pardue, H., *Clinical Chemistry* 1977, 23, 2189.
[8] Horne, T., *Abstracts from X International Congress of Clinical Chemistry,* Mexico 1978, 57, Abstr. No. 4.

### An automatic system for kinetic clinical analyses

**Clifford Riley**
*Royal Sussex County Hospital, Brighton, U.K.*
**Colin F. Darby**
*Bioengineering Department, University of Liverpool, Liverpool L69 3BX, U.K.*
**David E. Tutt**
*University of Sussex, Falmer, Brighton, U.K.*
**Bernard F. Rocks**
*Royal Sussex County Hospital, Brighton, U.K.*

THERE is a considerable need for an analytical system so designed that the relatively simple tests needed urgently in hospital may be carried out quickly at short notice. The following describes a possible solution to this problem.

Such a system should be capable of performing tests extremely rapidly with a precision at least as good as that found with existing conventional machines. The 'steady state' methods normally employed in analytical machines are for the most part too time consuming to suit an emergency system; the need is to make measurements in a matter of thirty seconds or so, a requirement which can be met by using reaction kinetics. The merits of kinetic methods have been reviewed by Pardue [1], Malmstadt et al [2] and Moss [3] and the measurement in blood plasma of a number of substances, other than enzymes, using such methods have been described; creatinine, (Cook [4] and Fabiny et al [5]), cholesterol, (Hewitt et al [6]), glucose, (Kadish [7]), urea, (Hallett et al [8]), thyroxin-eiodine, (Areq et al [9]) and amylase, (Shipe et al [10]). There is little doubt that kinetic methods can be at least as precise as traditional methods and indeed possess intrinsic advantages such as the reduced need to run blanks and short analysis time.

It is essential that such equipment can be left on standby for prolonged periods. Fortunately, modern opto-electronic systems are very stable and data can be stored indefinitely in digital registers.

An additional important requirement for such equipment is that it should be easy to use, so that if necessary, it will give precise

